# Global research trend and hotspot in the low FODMAP diet: a bibliometric analysis

**DOI:** 10.1186/s41043-024-00567-7

**Published:** 2024-05-13

**Authors:** Cheng Xu, Zhen Song, Jing-yi Hu, Chong-chao Li, Hong Shen

**Affiliations:** 1 Affiliated Hospital of Nanjing University of Chinese Medicine, Nanjing, China; 2 Nanjing University of Chinese Medicine, Nanjing, China

**Keywords:** FODMAP, Low FODMAP diet, Irritable bowel syndrome, Dietary management strategy, Bibliometric analysis, Hotspots

## Abstract

**Background:**

According to national guidelines, a diet low in fermentable oligosaccharides, disaccharides, monosaccharides, and polyols (FODMAP) is a second-line therapy option for irritable bowel syndrome (IBS) and improves functional intestinal symptoms. Numerous noteworthy results have been published in this field over the past fifteen years. This study aims to analyze the global research trend and hotspot of the low FODMAP diet research, and provide a comprehensive perspective and direction for researchers.

**Methods:**

The Science Citation Index-Expanded of the Web of Science Core Collection (WoSCC) was used to identify low FODMAP diet-related articles and reviews. Three bibliometric programs (CiteSpace, VOSviewer, Scimago Graphic) were utilized to analyze and visualize the annual publications, authors, countries, institutions, journals, citations, and keywords.

**Results:**

In total, 843 documents related to the low FODMAP diet research were published in 227 journals by 3,343 authors in 1,233 institutions from 59 countries. The United States, which was the most engaged nation in international collaboration, had the largest annual production and the fastest growth. The most productive organization was Monash University, and the most fruitful researcher was Gibson PR. *Nutrients* ranked first in terms of the number of published documents. The article “A diet low in FODMAPs reduces symptoms of irritable bowel syndrome” (Halmos EP, 2014) received the most co-citations. Keywords that appear frequently in the literature mainly involve two main aspects: the clinical efficacy evaluation and mechanism exploration of the low FODMAP diet. The term “gut microbiota” stands out as the most prominent keyword among the burst keywords that have remained prevalent till date.

**Conclusion:**

The restriction stage of the low FODMAP diet is superior to other dietary therapies for IBS in terms of symptom response, but it has a negative impact on the abundance of gut *Bifidobacteria* and diet quality. Identification of biomarkers to predict response to the low FODMAP diet is of great interest and has become the current research hotspot.

**Supplementary Information:**

The online version contains supplementary material available at 10.1186/s41043-024-00567-7.

## Highlights

The low FODMAP diet is the most recommended dietary intervention for the clinical management of IBS symptoms.

Bibliometrics analysis provides a comprehensive perspective and direction on global research trend and hotspot in the low FODMAP diet research.

The restriction stage of the low FODMAP diet is superior to other dietary therapies for IBS in terms of symptom response, but it has a negative impact on the abundance of gut *Bifidobacteria* and diet quality.

Identification of biomarkers to predict response to the low FODMAP diet has become the current research hotspot.

Large, well-designed clinical research studies are needed in the future to investigate the long-term efficacy and safety of the low FODMAP diet, including FODMAP reintroduction and personalization stages.

**Supplementary Information:**

The online version contains supplementary material available at 10.1186/s41043-024-00567-7.

## Introduction

Fermentable oligosaccharides, disaccharides, monosaccharides, and polyols (FODMAP) are a category of possibly poorly digested and absorbed but fermentable carbohydrates that can cause gastrointestinal discomfort in some people. Professor Gibson PR initially introduced the concept of the low FODMAP diet in 2005 for the prevention and treatment of Crohn’s disease [1], but the main focus of research quickly shifted to irritable bowel syndrome (IBS). IBS is a functional bowel disorder, mainly manifested by abdominal pain and bloating, accompanied by changes in bowel habits and/or abnormal stool characteristics [2]. The low FODMAP diet caused a short-term clinical response in 50–80% of IBS patients [3], and meta-analyses revealed that the diet was superior to other dietary therapies in terms of improving abdominal pain, bloating or distension, and bowel habits [4]. The low FODMAP diet was recommended as second-line therapy by national guidelines in United States [5, 6] and the United Kingdom [7, 8], and was perhaps the most evidence-based dietary intervention for IBS [5]. The low FODMAP diet is a therapeutic approach that includes an initial phase of restricting high FODMAP foods, followed by a systematic reintroduction process to evaluate an individual’s tolerance to different FODMAP categories. This step-wise approach ultimately leads to the development of a personalized diet plan tailored to each individual’s specific needs and tolerances [9].

Over the past fifteen years, there has been a significant increase in published studies on the low FODMAP diet. With such rapid growth, it becomes essential to adopt new approaches to review and interpret research trends. Bibliometrics, which combines mathematics, statistics, and philology, provides a multidimensional quantitative analysis and evaluation of literature, enabling a comprehensive understanding of the current state, potential development trend, and hotspot in a research area [10, 11]. To date, no bibliometric analysis has been conducted to assess the global research trend and hotspot in the low FODMAP diet. This study aims to address the following research questions by providing a comprehensive analysis through the examination of pertinent data collected from previous research related to low FODMAP diet research (Table ​1).

**Table 1:** Research questions

	Research Question	Objective	Motivation
1	Which authors, countries, institutions, and journals lead the research on the low FODMAP diet?	To identify the current landscape of published research	To better understand leadership and collaboration in this field
2	Which articles are co-cited the most, and which keywords appear most frequently in the literature?	To indicate which directions are of most concern to researchers	To provide insights into the knowledge base of the field
3	Which keywords are the burst keywords in recent years in the low FODMAP diet research?	To recognize the research focus that demonstrate a significant increase in occurrence during a specified period	To explore the frontier of low FODMAP diet research and predict future research hotspot

## Methods

### Data collection

**Fig. 1:**
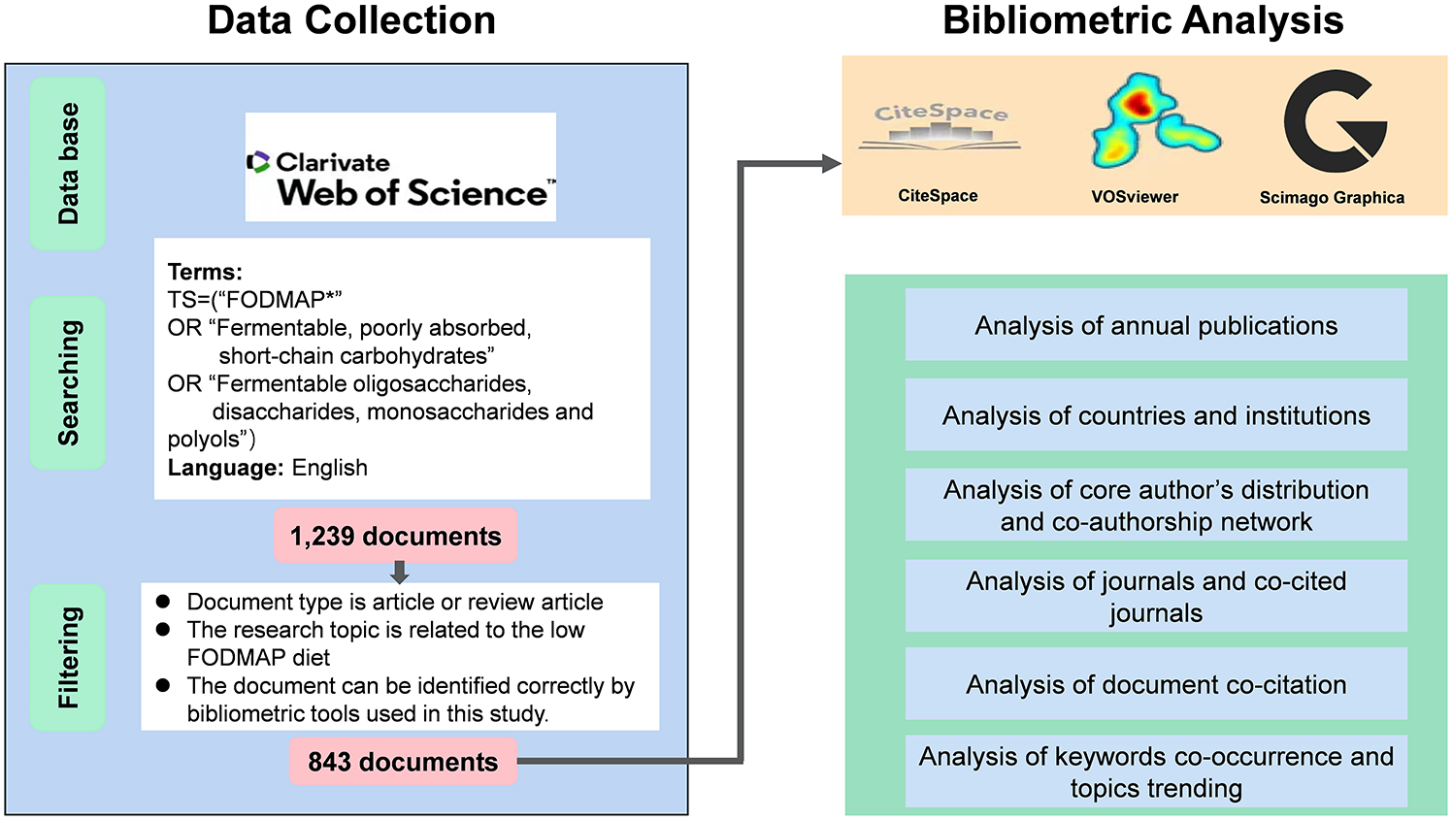
Flowchart for data collection and bibliometric analysis

The Science Citation Index-Expanded of the Web of Science Core Collection (WoSCC) database was utilized for the search. Two authors independently conducted and verified all searches on July 1, 2023, to ensure accuracy and eliminate bias resulting from database upgrades. The search strategy was as follows: TS= (“FODMAP*” OR “Fermentable, poorly absorbed, short-chain carbohydrates” OR “Fermentable oligosaccharides, disaccharides, monosaccharides and polyols”). Taking into account the constant updating of the database, we conducted a secondary search on April 13, 2024 to integrate the results of recently published studies that met the criteria. The search encompassed the entire time range from the establishment of the database to April 13, 2024, and was limited to publications written in English. Articles and review articles related to the low FODMAP diet that could be correctly identified using bibliometric tools were included in the bibliometric analysis. The detailed search plan and data filtering procedure are displayed in Fig. 1.

### Bibliometric analysis

The full record and cited references of all literature obtained from WoSCC were downloaded in TXT format [12]. The TXT files were then imported into CiteSpace (V6.3 R1 Advanced), VOSviewer (V1.6.19), and Scimago Graphica (V1.0.40) for further analysis [13–15].

CiteSpace, VOSviewer, and Scimago Graphica are commonly used bibliometric and visualization software platforms [16]. CiteSpace is a Java program created by Professor Chaomei Chen to analyze and visualize trends and patterns in scientific publications [17]. In this study, CiteSpace was used for dual-map overlap analysis of journals, document co-citation analysis, and keyword-related analysis and visualization. VOSviewer, a Java-based bibliometric mapping application developed by Leiden University, excelled in processing extensive bibliometric maps based on network data and presenting scientific information [18]. VOSviewer was utilized for co-occurrence analysis, identifying patterns among countries, institutions, authors, journals, and keywords. Scimago Graphica is an application designed to analyze and visualize data. Scimago Graphica was used to produce collaboration and geographic distribution maps of publications.

## Results

### Annual growth trend of publications

**Fig. 2:**
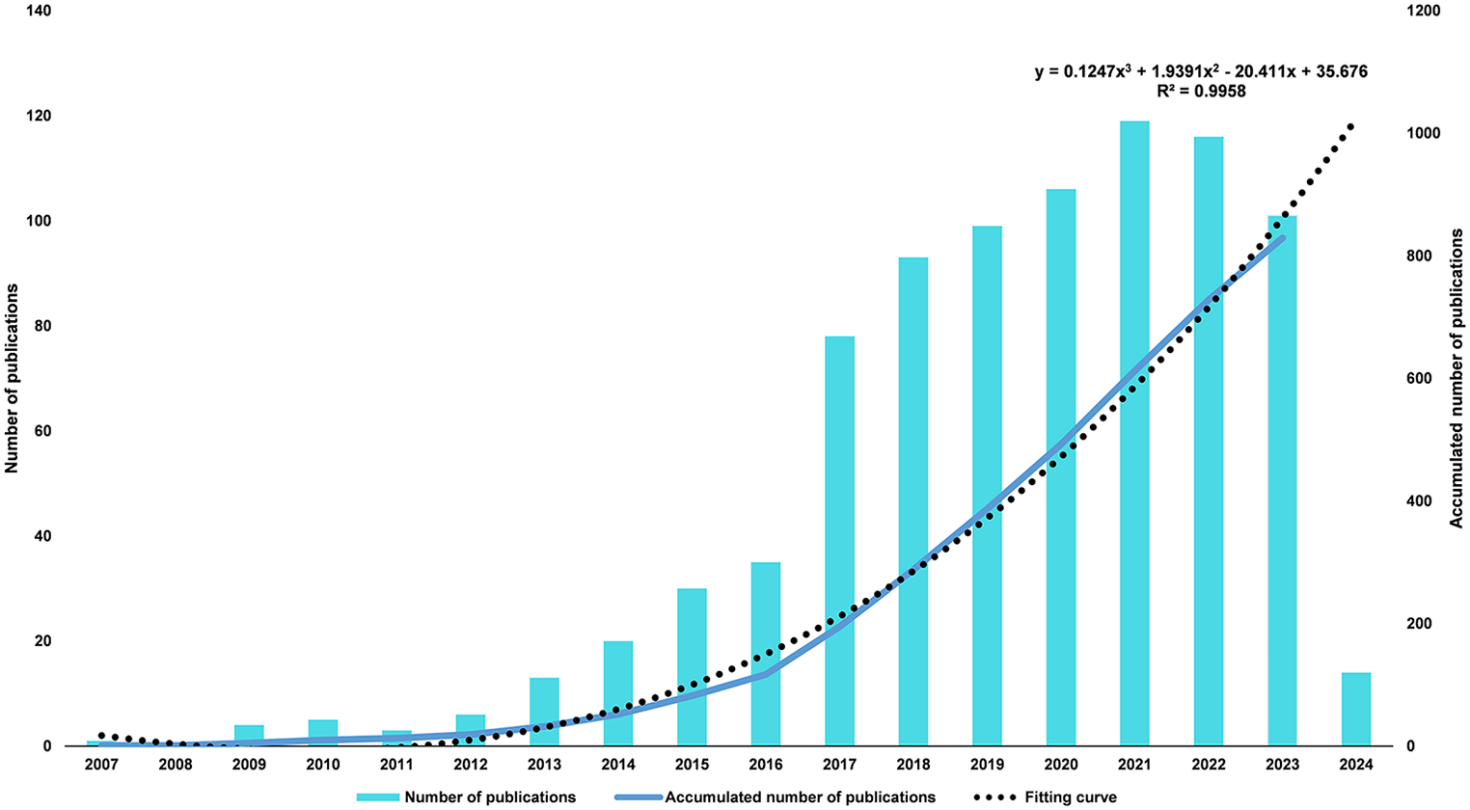
The number of annual research publications and growth trend related to low FODMAP diet research

A total of 843 publications related to the low FODMAP diet were identified through our search strategy, including 474 articles (56.23%) and 369 review articles (43.77%). The annual number of publications is displayed in Fig. 2. The number of publications in the early stage (2007–2012) remained small, with less than 10 publications per year. The subsequent four-year period (2013–2016) exhibited a steady growth pattern, with the annual publication count consistently exceeding twenty. Between 2017 and 2022, there was a considerable increase in the number of publications in this area, indicating that research on the low FODMAP diet has gained worldwide attention. However, there was a slight decline in the number of articles published in 2023. In general, judging from the fitting curve, the number of publications in this field will continue to grow steadily in the future.

### Distribution of countries and institutions

**Fig. 3:**
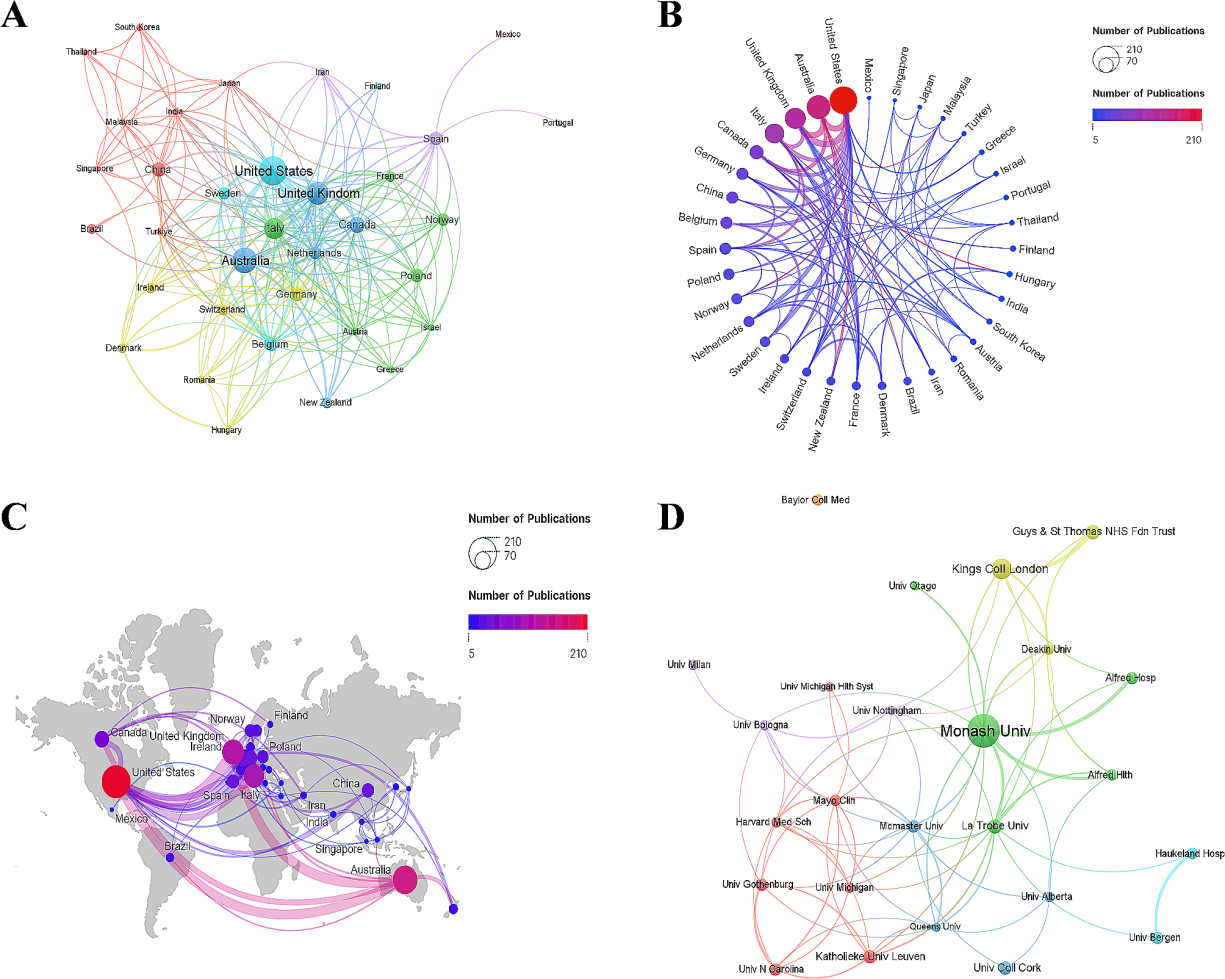
(**A**) Network map of the collaboration analysis of the low FODMAP diet research among countries, export of results from VOSviewer. Each node represents a country. The size of the node is proportional to the number of documents published. The lines between nodes represent cooperation between countries. (**B**) Visualization map of international collaboration generated by Scimago Graphica. The size of the node represents the number of publications, and the color and thickness of the line represents the strength of cooperation between countries. (**C**) Map of geographical distribution of publications generated by Scimago Graphica. The size of the node represents the number of publications, and the color and thickness of the line represents the strength of cooperation between countries. (**D**) Network map of the collaboration analysis of the low FODMAP diet research among institutions, export of results from VOSviewer. Each node represents an institution. The size of the node is proportional to the number of documents published. The lines between nodes represent cooperation between institutions

In total, 1,233 institutions from 59 different countries participated in the research of the low FODMAP diet. Table 2 lists the top 10 productive countries for scientific research. Most publications were produced in the United States (210, 24.91%), followed by Australia (155, 18.39%), the United Kingdom (120, 14.23%), Italy (105, 12.46%), and Canada (53, 6.29%). The United States had the highest annual output and the fastest growth, gradually overtaking Australia, which once maintained its leading position in the field. The United States was the most active country in international cooperation (Fig. 3A), and Australia and the United Kingdom had the most common cooperation with the United States (Fig. 3B and C). The top 10 productive institutions are listed in Table 2, with Monash University (99, 11.71%) in Australia ranking first, followed by King’s College London (39, 4.63%), La Trobe University (23, 2.73%), and Guy’s and St Thomas’ NHS Foundation Trust (21, 2.49%). The cooperation between institutions is depicted in Fig. 3D, with Monash University serving as the center of inter-institutional cooperation.

**Table 2:** Top 10 productive countries and top 10 productive institutions related to the low FODMAP diet research

Rank	Country	Count (% of 843)	Rank	Institution	Count (% of 843)	Location
1	United States	210 (24.91%)	1	Monash University	99 (11.71%)	Australia
2	Australia	155 (18.39%)	2	King’s College London	39 (4.63%)	United Kingdom
3	United Kingdom	120 (14.23%)	3	La Trobe University	23 (2.73%)	Australia
4	Italy	105 (12.46%)	4	Guy’s and St Thomas’ NHS Foundation Trust	21 (2.49%)	United Kingdom
5	Canada	53 (6.29%)	5	Catholic University of Leuven	20 (2.37%)	Belgium
6	Belgium	39 (4.63%)	6	University College Cork	20 (2.37%)	Ireland
7	China	39 (4.63%)	7	Alfred Hospital	18 (2.14%)	Australia
8	Germany	39 (4.63%)	8	University of Bergen	18 (2.14%)	Norway
9	Spain	38 (4.51%)	9	University of North Carolina	18 (2.14%)	United States
10	Poland	34 (4.03%)	10	McMaster University	16 (1.90%)	Canada

### Core author’s distribution and co-authorship network

**Fig. 4:**
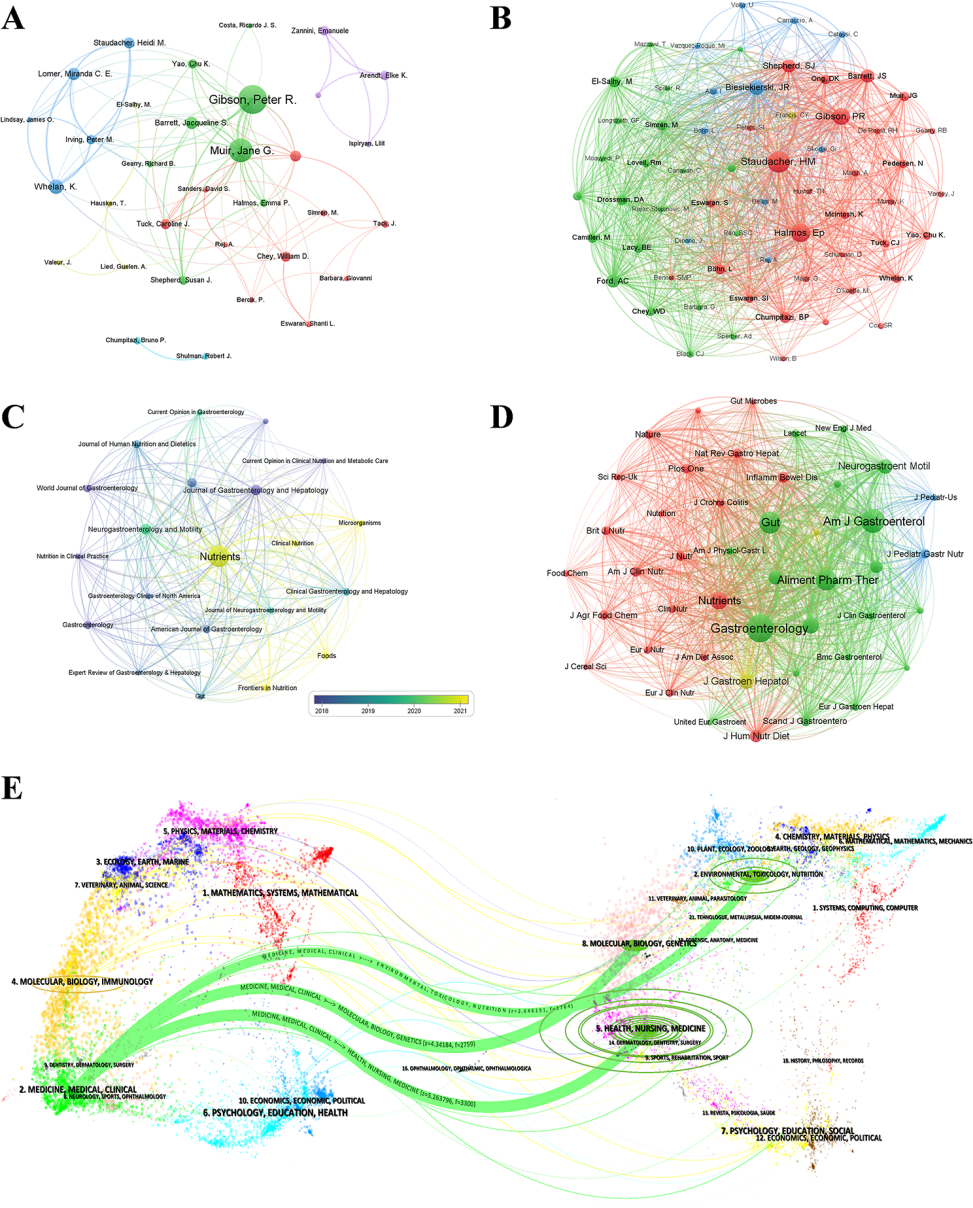
(**A**) Network map of the collaboration analysis of low FODMAP diet research among core authors, export of results from VOSviewer. Each node represents an author. The size of the node is proportional to the number of documents published. (**B**) Network map of the co-cited authors related to low FODMAP diet research, export of results from VOSviewer. The size of the node is proportional to the number of citations. (**C**) Network map of the academic journals publishing low FODMAP diet research, export of results from VOSviewer. Each node represents a journal. The size of the node is proportional to the number of documents published. The colors represent the average year of publications (blue: earlier, yellow: later). (**D**) Network map of the co-cited journals publishing low FODMAP diet research, export of results from VOSviewer. The size of the node is proportional to the number of citations. (**E**) A dual-map overlap of journals publishing low FODMAP diet research, export of results from CiteSpace. The left side is the citing journal, the right side is the cited journal, and the longer transverse width of the ellipse indicates more documents in the relevant journal

In all, 3,343 authors contributed to research on the low FODMAP diet. Table S1 lists the top 10 authors in terms of publications and co-citations. The top three authors based on the number of publications were Gibson PR (65, 7.71%), Muir JG (44, 5.22%), and Whelan K (29, 3.44%). The network visualization map offers vivid details of cooperative interactions, aiding in the identification of possible partnerships (Fig. 4A). In terms of co-citations, Staudacher HM had the most citations, reaching 909, followed by Halmos EP (711), and Gibson PR (607). Co-citation relationships occur when two publications are jointly cited by a third citation publication [19]. The network visualization of the relationship between co-cited authors is displayed in Fig. 4B. Four of the top 10 productive authors were among the top 10 co-cited authors (Gibson PR, Staudacher HM, Biesiekierski JR, and Barrett JS).

### Analysis of journals and co-cited journals

Publications related to the low FODMAP diet research were found in 227 journals. The top 10 productive journals and co-cited journals related to the low FODMAP diet research are listed in Table 3. As shown in Fig. 4C, *Nutrients* had the greatest volume with 140 documents (14.61%), followed by *Neurogastroenterology and Motility* (32, 3.80%), *Journal of Gastroenterology and Hepatology* (31, 3.68%), and *Alimentary Pharmacology & Therapeutics* (24, 2.85%). The frequency of co-citations, which indicates a journal’s substantial effect on a certain topic, is a key indicator of its influence. When ranked by co-citations, the top three journals were *Gastroenterology* (4174), *American Journal of Gastroenterology* (3166), and *Gut* (2642). The network visualization of co-cited journals is shown in Fig. 4D. Among the top 10 co-cited journals, 40% were in the United Kingdom and 30% were in the United States. Additionally, 90% of these journals belonged to the Q1 or Q2 JCR division. The topical distribution of academic journals is depicted in the dual-map overlay of journals (Fig. 4E). The colored paths show the citation relationships, with the citing journals on the left and the cited journals on the right. As shown, the low FODMAP diet research was mainly published in journals about “medicine, medical, clinical” subjects, and the documents cited by these studies were mostly published in journals related to “environmental, toxicology, nutrition”, “molecular, biology, genetics” or “health, nursing, medicine subjects”.

**Table 3:** Top 10 productive journals and co-cited journals related to the low FODMAP diet research

Rank	Journals	Count (% of 843)	JCR (2022)	Rank	Co-cited journals	Co-citation	JCR (2022)
1	Nutrients (Switzerland)	140 (14.61)	Q1	1	Gastroenterology (United States)	4174	Q1
2	Neurogastroenterology and Motility (United Kingdom)	32 (3.80)	Q2	2	American Journal of Gastroenterology (United States)	3166	Q1
3	Journal of Gastroenterology and Hepatology (Australia)	31 (3.68)	Q2	3	Gut (United Kingdom)	2642	Q1
4	Alimentary Pharmacology & Therapeutics (United Kingdom)	24 (2.85)	Q1	4	Alimentary Pharmacology & Therapeutics (United Kingdom)	2549	Q1
5	Journal of Human Nutrition and Dietetics (United Kingdom)	18 (2.14)	Q3	5	Nutrients (Switzerland)	1761	Q1
6	American Journal of Gastroenterology (United States)	17 (2.02)	Q1	6	Clinical Gastroenterology and Hepatology (United States)	1445	Q1
7	Gastroenterology (United States)	17 (2.02)	Q1	7	Journal of Gastroenterology and Hepatology (Australia)	1297	Q2
8	Clinical Gastroenterology and Hepatology (United States)	16 (1.90)	Q1	8	Neurogastroenterology and Motility (United Kingdom)	1266	Q2
9	World Journal of Gastroenterology (China)	15 (1.78)	Q2	9	World Journal of Gastroenterology (China)	1067	Q2
10	Foods (Switzerland)	13 (1.54)	Q1	10	Journal of Human Nutrition and Dietetics (United Kingdom)	887	Q3

### Analysis of document co-citation

A total of 28,797 references were cited in the 843 included documents. Table 4 lists the top 10 highly co-cited documents related to research on the low FODMAP diet, all of which were clinical trials investigating the clinical efficacy of this therapy. The most highly co-cited document, authored by Halmos EP, received 217 citations. Among the top 10 highly co-cited documents, Halmos EP and Staudacher HM each had two documents. All of the top 10 highly co-cited documents were published in JCR Q1 or Q2 journals, with four of them published in *Gastroenterology* and two in *Gut*.

**Table 4:** Top 10 highly co-cited documents related to the low FODMAP diet research

Rank	Title	First author	Year	Citation		Journal	DOI	JCR (2022)
1	A diet low in FODMAPs reduces symptoms of irritable bowel syndrome	Halmos EP	2014	217		Gastroenterology	10.1053/j.gastro.2013.09.046	Q1
2	Diet low in FODMAPs reduces symptoms of irritable bowel syndrome as well as traditional dietary advice: a randomized controlled trial	Bohn L	2015	196		Gastroenterology	10.1053/j.gastro.2015.07.054	Q1
3	Diets that differ in their FODMAP content alter the colonic luminal microenvironment	Halmos EP	2015	196		Gut	10.1136/gutjnl-2014-307264	Q1
4	Fermentable carbohydrate restriction reduces luminal bifidobacteria and gastrointestinal symptoms in patients with irritable bowel syndrome	Staudacher HM	2012	150		The Journal of Nutrition	10.3945/jn.112.159285	Q2
5	FODMAPs alter symptoms and the metabolome of patients with IBS: a randomised controlled trial	McIntosh K	2017	150		Gut	10.1136/gutjnl-2015-311339	Q1
6	A Diet Low in FODMAPs Reduces Symptoms in Patients With Irritable Bowel Syndrome and A Probiotic Restores Bifidobacterium Species: A Randomized Controlled Trial	Staudacher HM	2017	148		Gastroenterology	10.1053/j.gastro.2017.06.010	Q1
7	A Randomized Controlled Trial Comparing the Low FODMAP Diet vs. Modified NICE Guidelines in US Adults with IBS-D	Eswaran SL	2016	144		American Journal of Gastroenterology	10.1038/ajg.2016.434	Q1
8	No effects of gluten in patients with self-reported non-celiac gluten sensitivity after dietary reduction of fermentable, poorly absorbed, short-chain carbohydrates	Biesiekierski JR	2013	135	Gastroenterology		10.1053/j.gastro.2013.04.051	Q1
9	Manipulation of dietary short chain carbohydrates alters the pattern of gas production and genesis of symptoms in irritable bowel syndrome	Ong DK	2010	125	Journal of Gastroenterology and Hepatology		10.1111/j.1440-1746.2010.06370.x	Q2
10	Randomised clinical trial: gut microbiome biomarkers are associated with clinical response to a low FODMAP diet in children with the irritable bowel syndrome	Chumpitazi BP	2015	123	Alimentary Pharmacology & Therapeutics		10.1111/apt.13286	Q1

### Analysis of keywords co-occurrence and topics trending

**Fig. 5:**
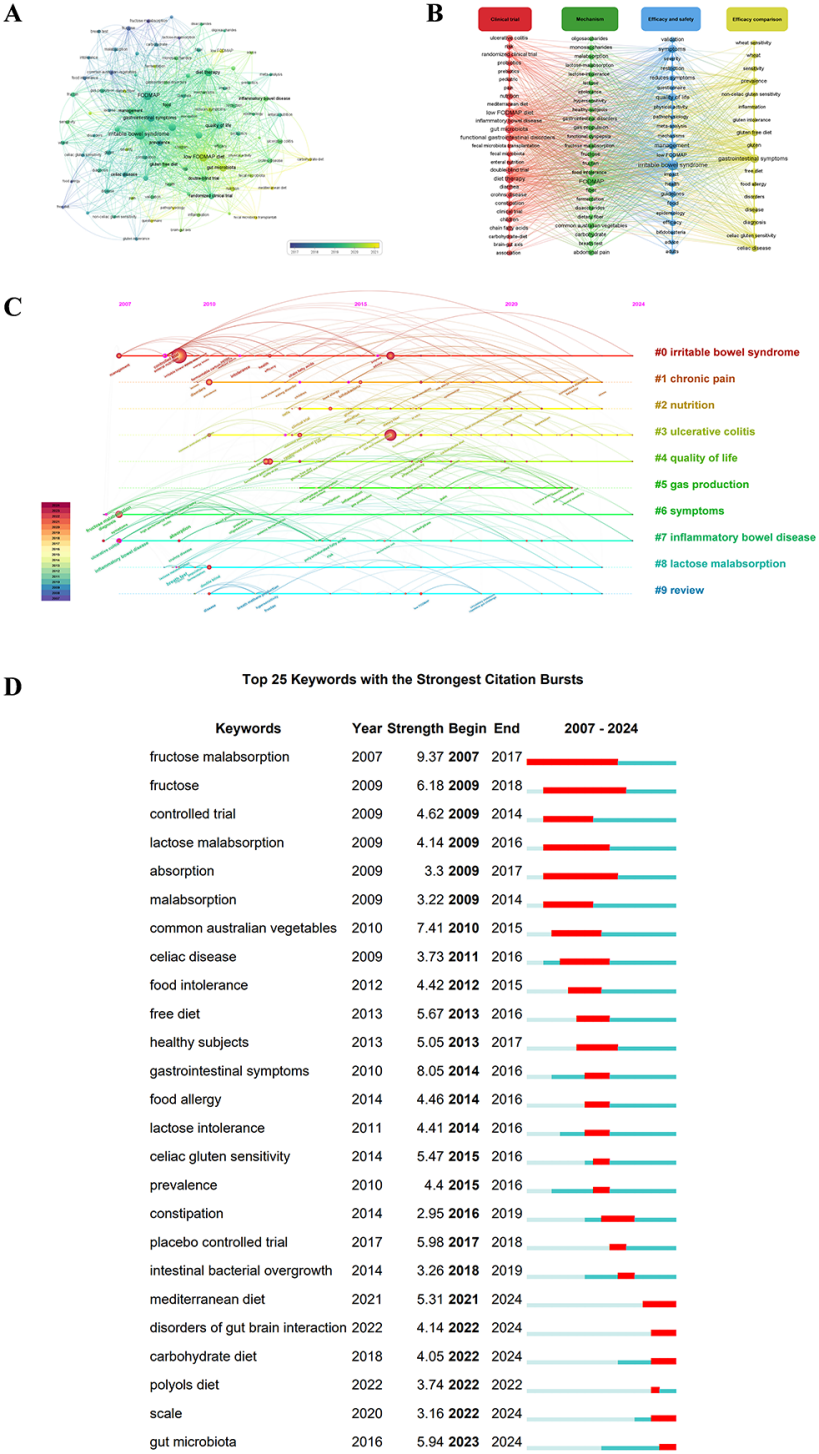
(**A**) Temporal view of keywords co-occurrence generated by VOSviewer. Each node represents a keyword. The size of each node is proportional to the frequency of occurrence. The colors represent the average year of keyword occurrence (blue: earlier, yellow: later). (**B**) Clustering visual analysis map of keywords generated by VOSviewer. The size of each node is proportional to the frequency of occurrence. Nodes of different colors form separate clusters, each representing distinct research directions. (**C**) Timeline view of keywords co-occurrence generated by CiteSpace. Each node represents a keyword. The node size, indicative of occurrence frequency, correlates with the sum of their sizes along the yearly ring line. The links between keywords indicate co-occurrence, where deep blue signifies earlier appearances, deep red represents later ones, and overlapping colors denote occurrences in corresponding years. (**D**) Keywords with the strongest burst strength related to low FODMAP diet research during the period of 2007–2024. The burst period is represented by the red section on the blue timeline, export of results from CiteSpace

Keywords play a crucial role in revealing the primary themes of an academic publication. Fig. 5A depicts the co-occurrence analysis of keywords, and Table S2 lists the top 20 keywords by frequency. Among the 459 keywords originating from the 843 included documents, the three most frequently used keywords were “irritable bowel syndrome” (529), “low FODMAP diet” (314), and “gastrointestinal symptoms” (216). The clustering visual analysis map delineates four principal research topics within the realm of low FODMAP diet, encompassing clinical trials, mechanisms, efficacy and safety, and efficacy comparison (Fig. 5B).

The hierarchical cluster labeling method was employed to identify keywords most relevant to research on the low FODMAP diet. As shown in Fig. 5C, these keywords were grouped into ten clusters, primarily focusing on evaluating the clinical efficacy (#0 irritable bowel syndrome, #3 ulcerative colitis, #4 quality of life, #6 symptoms, #7 inflammatory bowel disease) and exploring the mechanisms (#1 chronic pain, #2 nutrition, #9 gas production, #8 lactose malabsorption) of the low FODMAP diet. The clustering timeline view combines cluster analysis with time slice analysis to provide a clear depiction of the distribution and trend of keywords over time.

Keyword burst detection is a technique for detecting topics trending and current hotspot. The top 25 keywords with the strongest burst strength related to the low FODMAP diet research from 2007 to 2024 are presented in Fig. 5D. Prior to 2012, there was a significant emergence of burst keywords such as “fructose malabsorption”, “lactose malabsorption”, and “common Australian vegetables”. Subsequently, from the period of 2013 to 2021, keywords like “gastrointestinal symptoms”, “placebo-controlled trial”, and “healthy subjects” exhibited a high burst strength. The recent burst in keywords such as “gut microbiota” (with a burst strength of 5.94), “mediterranean diet” (with a burst strength of 5.31), “disorders of gut brain interaction” (with a burst strength of 4.14), “carbohydrate diet” (with a burst strength of 4.05), and “scale” (with a burst strength of 3.16) reflects the emerging trends in the low FODMAP diet research. Among these keywords, “gut microbiota” stands out with the highest burst strength, signifying its position as the research hotspot in this field.

## Discussion

### General information

The low FODMAP diet has gained significant attention in recent years within the scientific literature. This article presents the first study to utilize the bibliometric method for analyzing documents related to low FODMAP diet research, employing mathematical statistics and visual analysis to identify development trend and research hotspot based on data information.

Over the past fifteen years, there has been an increase in research related to the low FODMAP diet. Dietary therapy has become an important part of multidisciplinary intestinal disease management, evolving from a virtually non-existent position in the mid-20th century to one oriented on patient care and now plays an essential role in providing treatment [20, 21]. Furthermore, the increasing research on the low FODMAP diet can be attributed to its value in controlling functional gastrointestinal symptoms, particularly in IBS. It is currently considered the most evidence-based dietary intervention for IBS [5], and has been recommended as a second-line herapy option for IBS [8, 22, 23]. The increase in studies demonstrates the prospective future for the low FODMAP diet research and requires greater focus and in-depth investigation.

The United States has emerged as the leading country in terms of publications in the field of low FODMAP diet research. This is unsurprising considering its academic funding and economic growth [24]. In addition to the United States, Australia, and the United Kingdom have published more than 100 documents related to the low FODMAP diet research, reflecting the fact that current studies are based on western dietary patterns. Among institutions, Monash University in Australia had the most publications worldwide. The study team from Monash University, a pioneer in this field, was the first to propose and develop the low FODMAP diet [1]. The United States and Australia exhibit the highest level of cooperation, which is unsurprising considering the United States’ significant output and Australia’s role as the initiator of low FODMAP diet research. The cooperation between the European region, represented by the United Kingdom, and the United States or Australia should not be ignored. In general, the research on the low FODMAP diet is a domain of global cooperation, so it is very important to strengthen institutional and international collaboration to promote the sustainable development of this field.

The number of publications represents the author’s contribution to the research field, and the number of citations reflects the author’s influence. Among the authors who contributed to the research of the low FODMAP diet from 2007 to 2024, Gibson PR from Monash University published the most documents. Gibson PR’s research team has been at the forefront of investigating the mechanisms and clinical aspects of this therapy. Staudacher HM from Deakin University is the most co-cited author in this field, focusing on researching the impact of the low FODMAP diet on gut microbiota, and relevant research literature has been widely cited [25, 26]. Gibson PR, Staudacher HM, Biesiekierski JR, and Barrett JS are among the top 10 authors in terms of both published documents and citations, indicating their extensive scientific output and significant influence.

Analysis of journal publishing volume and journal co-citations can demonstrate their contributions to the field, and researchers can utilize these results to identify appropriate journals for submission. Among the 227 journals that have published low FODMAP diet research, *Nutrients* stands out with the highest number of publications, totaling 140. *Nutrients* is an international journal focused on nutrition and human health, and its considerable influence in the low FODMAP diet research field is noteworthy. It has emerged as a prominent platform for the dissemination of the latest and most extensive research in this area. Besides, documents published in *Gastroenterology* occupied the highest citation. As the official journal of the American Gastroenterological Association, *Gastroenterology* holds a prestigious position within the field of gastrointestinal diseases, demonstrating that some high-level and influential journals value the low FODMAP diet research. The results of the dual-map overlap of journals revealed that the majority of low FODMAP diet research was published in “medicine, medical, clinical” journals, and the cited literature was mostly published in “environmental, toxicology, nutrition”, “molecular, biology, genetics” or “health, nursing, medicine” journals, indicating that the research in this field was mainly focused on clinical trials and translational medicine research.

### Knowledge base

Highly cited publications serve as indicators of the research hotspot within the field, and analyzing the cited references provides insights into the knowledge base of the field. Notably, the top 10 highly co-cited documents are all randomized controlled trials (RCTs) of the low FODMAP diet, serving as reliable reference resources for future research in this area. The first RCT of the low FODMAP diet found that after 4 weeks of restriction of fermentable carbohydrates, it significantly relieved the symptoms of IBS compared to a habitual diet [25]. Subsequent studies have consistently confirmed the efficacy of the low FODMAP diet. Most of these studies demonstrated that the low FODMAP diet had a positive effect on IBS symptoms compared to habitual diets [27], typical diets [28, 29], a high FODMAP diet [30], or a sham diet [26], and several studies found its equivalent treatment efficacy to standard dietary advice [31] or general dietary guidelines [32]. However, the quality of evidence was rated as low due to potential bias stemming from a lack of double-blinding and inadequate reporting of suboptimal adverse events [4]. It is important to note that while the efficacy of the low FODMAP diet is considered “low quality evidence” according to GRADE criteria, it is recognized that dietary interventions rarely meet the criteria for “high quality evidence” used to evaluate pharmaceutical trials [33]. Several management guidelines have still recommended the use of this diet as a primary or secondary treatment for IBS [6, 34].

Diet exerts a significant influence on the human gut microbiota [35, 36], and the low FODMAP diet is no exception. Several studies among the top 10 highly co-cited documents have reported a relative reduction in total bacterial abundance due to FODMAP restriction, including a decrease in gut microbes typically linked with health, such as *Bifidobacterium* [25, 26, 37]. The first RCT of the low FODMAP diet showed a decrease in the proportion and concentration of luminal *Bifidobacteria* compared to a habitual diet [25]. Similarly, two other RCTs demonstrated a lower absolute abundance of *Bifidobacteria* in the low FODMAP diet compared to a placebo diet [26, 37]. In contrast, another RCT did not observe a decrease in *Bifidobacteria* with the low FODMAP diet, but rather found an increase in the *Bifidobacteriaceae* family and certain species within the family *Lachnospiraceae* when following a high FODMAP diet [30]. The consistent finding of reduced *Bifidobacteria* abundance due to the low FODMAP diet raises concerns about potential adverse consequences, although the health effects of lower *Bifidobacteria* resulting from this diet remain unknown. In the long term, the adverse effects on luminal *Bifidobacteria* levels caused by FODMAP restriction can be effectively restored through FODMAP personalization [38], emphasizing the essential role played by the reintroduction and personalization stages in the low FODMAP diet [39]. However, further rigorous clinical trials are still necessary to establish the long-term efficacy and safety of the low FODMAP diet.

### Research trend and hotpot

Visualized analysis of keywords reveals the evolution of high-frequency keywords and shows the development path of the low FODMAP diet. After clustering, we obtained 10 clustering labels that encompassed two primary aspects: the evaluation of clinical efficacy (#0 irritable bowel syndrome, #2 ulcerative colitis, #4 functional dyspepsia, #5 children, #6 celiac disease, #7 dietary interventions, #8 diet quality) and the exploration of mechanisms (#0 gas production, #2 colonic fermentation, #9 bifidobacteria) related to the low FODMAP diet.

Clinical research on the low FODMAP diet has mostly focused on its efficacy in IBS, which has been discussed in the previous sections. However, it is exciting to explore the potential efficacy of the low FODMAP diet for diseases other than IBS. More and more research data supported the use of the low FODMAP diet in conditions such as inflammatory bowel disease [40, 41], functional dyspepsia [42–44], and celiac disease [45], etc. The low FOAMAP diet can assist in symptom management of a variety of diseases, which is thought to be related to the underlying pathological mechanism of FODMAP’s involvement in visceral hypersensitivity [46]. While encouraging, larger and more rigorously designed clinical trials evaluating the long-term effects of the low FODMAP diet are needed to evaluate its efficacy and safety in clinical practice.

The low FODMAP diet is the most recommended dietary intervention for managing of IBS symptoms, but faces challenges in dietary therapy development. In addition to its impact on gut microbiota mentioned above, the low FODMAP diet may also affect nutrition intake and diet quality. Patients with IBS were reported to have lower calcium intakes than those who followed a regular diet after following the low FODMAP diet for 4 weeks [25], as well as lower calorie, carbohydrate, and fiber intakes when compared to those following the diet recommended by the National Institute for Health and Care Excellence [32]. While the implementation of the low FODMAP diet was observed to reduce several micronutrients, most of these reductions were not significant after adjusting for energy intake, except for riboflavin [47]. An RCT of 130 individuals revealed that the low FODMAP diet had higher intakes of vitamin B_12_ and selenium than the sham diet and more intakes of vitamin B_12_ than a habitual diet, but decreased diet quality compared with the habitual control diet [48]. The reintroduction and personalization of FODMAP can be a solution to the nutritional deficiency that can occur with FODMAP restriction [49]. Besides, several attempts have been made to include dietary supplements to enhance the nutritional value of the diet, with specific supplements showing additional symptomatic benefits compared to FODMAP restriction alone [50, 51]. Clinicians and dietitians should provide guidance to optimize nutrient intake, maintain diet quality and enhance patient adherence [52, 53].

The low FODMAP diet may improve gastrointestinal symptoms through various mechanisms, and further research on mechanisms may broader its clinical application. FODMAP malabsorption leads to intestinal fermentation, gas production, and an increase in osmotic pressure, which stimulate mechano- and chemoreceptors, resulting in pain, decreased gastrointestinal motility, flatulence, and bloating [43]. However, a study using MRI showed that after consuming fermentable carbohydrates, IBS patients and healthy controls had comparable levels of gas and bowel distension, which suggested that the colonic hypersensitivity to distension, rather than the excess of gas, was the underlying cause of symptoms in IBS patients [54]. A recent finding has revealed that the low FODMAP diet can alter visceral hypersensitivity by increasing colon microcirculation perfusion and decreasing the expression of vascular endothelial-derived growth factor [55]. Another hypothesized mechanism by which FODMAPs cause gastrointestinal symptoms is related to increased histamine. It has been reported that IBS patients have increased urinary histamine levels and the low FODMAP diet can decrease histamine levels [30]. The cause of the histamine elevation has not been identified, as it may derive from dietary sources, or be produced by colonic mast cells or intestinal microbiota [56]. In addition, a decrease in the inflammatory cytokines interleukin (IL)-6 and IL-8 have been reported in IBS patients following the low FODMAP diet [57]. Therefore, the low FODMAP diet may improve gastrointestinal symptoms by regulating mucosal barrier and proinflammatory factors.

Keyword burst is regarded as a key indicator of trend and hotspot in a research field. The period prior to 2012 witnessed the burst of keywords such as “fructose malabsorption”, “lactose malabsorption”, and “common Australian vegetables”, which served as markers of the formulation and development of new concept within the field. Subsequently, spanning from 2013 to 2021, burst keywords like “gastrointestinal symptoms”, “placebo controlled trial”, and “healthy subjects” indicated a substantial surge in clinical trials performed during this timeframe to assess the effects of the low FODMAP diet. Burst keyword that has persisted until now can be regarded as the forefront of the low FODMAP diet research. The low FODMAP diet can improve the clinical condition in 50-80% of IBS patients [3]. In other words, 20–50% of individuals do not respond to the low FODMAP diet. Therefore, research on identifying biomarkers to predict response to the low FODMAP diet has become a prominent topic. Predicting responses to the low FODMAP diet based on fecal bacteria profiles is an emerging research field. The fecal microbiota has been analyzed by a “GA-map Dysbiosis Test” to create a “Dysbiosis Index” score, which provides a numerical score indicating how an individual’s bacterial composition compares to a healthy reference population, with some bacteria having higher abundance than others, including *Bacteroides stercoris*, *Acinetobacter*, *Pseudomonas*, and genus *Desulfitispora* [58]. Children with IBS who responded to the low FODMAP diet had a higher abundance of certain bacteria at baseline, such as *Bacteroides*, *Ruminococcaceae*, and *Faecalibacterium prausnitzii*, which are known to have great saccharolytic metabolic capacity [29, 59]. Moreover, a recent study stratified IBS patients based on gut microbiota species and metabolic genetic characteristics, identifying two distinct microbiota profiles for IBS pathogenic-like and IBS health-like subtypes [60]. Patients with IBS pathogenic-like subtypes had a greater clinical response to the low FODMAP diet than those with IBS health-like subtypes [60]. However, a recent finding showed that the fecal microbiota did not predict response to the low FODMAP diet, and supported the distinction between the low FODMAP diet responders and non-responders based on fecal metabolites [61]. A previous study also used fecal volatile organic compounds at baseline to predict the response of IBS patients to the low FODMAP diet with 97% accuracy [62]. As a low-cost and non-invasive method, fecal volatile organic compounds profiling can be used to predict whether IBS patients would respond to the low FODMAP diet, but it still has to be verified by a large prospective cohort. The research on predictors of response to the low FODMAP diet is currently a hotspot, with preliminary evidence supporting the use of fecal microbiota or fecal metabolites. However, these methods need to be tested in larger external validation populations.

### Strengths and limitations

To our knowledge, this study is the first to comprehensively summarize and analyze the knowledge base, research trend and current hotspot of the low FODMAP diet research using bibliometrics. Compared with traditional literature reviews, a bibliometric analysis based on bibliometrics tools (CiteSpace, VOSviewer, Scimago Graphic) can provide a relatively comprehensive and objective presentation of the data to better describe and visualize the research trend and hotspot. However, it is important to acknowledge that this study has inherent limitations due to the use of bibliometric analysis. First, the WOSCC database is still being updated, and some of the updated documents were not included in our study, so the results could not fully reflect the situation of the documents published in 2024. Second, the documents included in our study may not be complete. On the one hand, we only focused on data from the WoSCC database since CiteSpace can only analyze and visualize co-citation maps of data retrieved from this database. This selection was made due to the unavailability of co-citation analysis support on other significant search engines such as PubMed, Embase, and Ovid. On the other hand, due to the uniformity of data extraction, only published English literature was searched, and some bias was introduced. Nevertheless, considering the authority of the WoSCC database and the widespread use of English as the predominant international language, we consider this study still effectively portrays the overall situation in this field. Third, since citations to documents take time to accumulate, their amount does not accurately reflect the influence of the documents. Early published literature may receive more citations, while newer high-quality publications may require more time to accumulate citations.

## Conclusion

This research is the first bibliometric analysis to summarize and visualize the development of the low FODMAP diet research, and explore the research trend and hotspot in this field. The gradual increase in published documents over the past fifteen years suggests that this field is receiving more attention from researchers. The research in this area has mainly focused on the evaluation of clinical efficacy and exploration of the mechanism of the low FODMAP diet in the treatment of IBS. The restriction stage of the low FODMAP diet is superior to other dietary therapies for IBS in terms of symptom response, but it has a detrimental influence on the abundance of gut *Bifidobacteria* and diet quality. Identification of biomarkers to predict response to the low FODMAP diet is of great interest and has become the current research hotspot. To provide higher levels of clinical evidence, large, well-designed clinical research studies are needed in the future to investigate the long-term efficacy and safety of the low FODMAP diet, including FODMAP reintroduction and personalization stages. We hope that this study will aid researchers in better comprehending the general trends in this field and can offer direction for further study.

### Electronic supplementary material

Below is the link to the electronic supplementary material.

Supplementary Material 1

## Data Availability

The data that support the findings of this study are available from the corresponding author upon reasonable request.

## Abbreviations

FODMAP: Fermentable oligosaccharides, disaccharides, monosaccharides, and polyols
IBS: Irritable bowel syndrome
WoSCC: Web of Science Core Collection
RCT: Randomized controlled trials
IL: Interleukin
