# Supplementary material for: Global research trend and hotspot in the low FODMAP diet: a bibliometric analysis
Source: J Health Popul Nutr. 2024 May 13;43:63. doi: 10.1186/s41043-024-00567-7 (PMC11092139; doi:10.1186/s41043-024-00567-7)
Supplement: Supplementary file 1 — Supplementary Material 1 [file 41043_2024_567_MOESM1_ESM.docx]

**Table S1** Top 10 productive authors and top 10 co-cited authors related to the low FODMAP diet research.

| Rank | Author | Count (% of 843) | Rank | Co-cited author | Co-citation |
| --- | --- | --- | --- | --- | --- |
| 1 | Gibson PR | 65 (7.71%) | 1 | Staudacher HM | 909 |
| 2 | Muir JG | 44 (5.22%) | 2 | Halmos EP | 711 |
| 3 | Whelan K | 29 (3.44%) | 3 | Gibson PR | 607 |
| 4 | Barrett JS | 20 (2.37%) | 4 | Biesiekierski JR | 463 |
| 5 | Biesiekierski JR | 20 (2.37%) | 5 | Shepherd SJ | 395 |
| 5 | Chey WD | 19 (2.25%) | 6 | Ford AC | 390 |
| 5 | Staudacher HM | 18 (2.14%) | 7 | Barrett JS | 362 |
| 8 | Arendt EK | 17 (2.02%) | 8 | Chumpitazi BP | 276 |
| 8 | Zannini E | 17 (2.02%) | 9 | El-Salhy M | 264 |
| 10 | Irving PM | 16 (1.90%) | 10 | Drossman DA | 248 |

**Table S2** Top 20 keywords with the highest count related to the low FODMAP diet research**.**

| Rank | Keywords | Count | Centrality | Rank | Keywords | Count | Centrality |
| --- | --- | --- | --- | --- | --- | --- | --- |
| 1 | irritable bowel syndrome | 529 | 0.01 | 11 | randomized clinical trial | 94 | 0.09 |
| 2 | low FODMAP diet | 314 | 0.01 | 12 | FOAMAP | 87 | 0.05 |
| 3 | gastrointestinal symptoms | 216 | 0.05 | 13 | inflammatory bowel disease | 87 | 0.15 |
| 4 | symptoms | 190 | 0.05 | 14 | gluten free diet | 83 | 0.04 |
| 5 | quality of life | 176 | 0.04 | 15 | celiac disease | 81 | 0.1 |
| 6 | gut microbiota | 160 | 0.01 | 16 | diet therapy | 72 | 0.03 |
| 7 | functional gastrointestinal disorders | 137 | 0.05 | 17 | efficacy | 72 | 0.03 |
| 8 | management | 131 | 0.09 | 18 | food | 72 | 0.01 |
| 9 | prevalence | 119 | 0.05 | 19 | ulcerative colitis | 71 | 0.04 |
| 10 | double blind | 115 | 0.04 | 20 | crohns disease | 70 | 0.01 |
